# Mitochondria in Focus: From Function to Therapeutic Strategies in Chronic Lung Diseases

**DOI:** 10.3389/fimmu.2021.782074

**Published:** 2021-11-23

**Authors:** Dayene de Assis Fernandes Caldeira, Daniel J. Weiss, Patricia Rieken Macêdo Rocco, Pedro Leme Silva, Fernanda Ferreira Cruz

**Affiliations:** ^1^ Laboratory of Pulmonary Investigation, Institute of Biophysics Carlos Chagas Filho, Federal University of Rio de Janeiro, Rio de Janeiro, Brazil; ^2^ Department of Medicine, College of Medicine, University of Vermont, Burlington, VT, United States; ^3^ National Institute of Science and Technology for Regenerative Medicine, Rio de Janeiro, Brazil; ^4^ Rio de Janeiro Innovation Network in Nanosystems for Health-NanoSAÚDE/FAPERJ, Rio de Janeiro, Brazil

**Keywords:** mitochondrial dysfunction, mitochondrial dynamics, mitochondrial morphology, mitotherapy, chronic lung diseases, reactive species of oxygen (ROS)

## Abstract

Mitochondria are essential organelles for cell metabolism, growth, and function. Mitochondria in lung cells have important roles in regulating surfactant production, mucociliary function, mucus secretion, senescence, immunologic defense, and regeneration. Disruption in mitochondrial physiology can be the central point in several pathophysiologic pathways of chronic lung diseases such as chronic obstructive pulmonary disease, idiopathic pulmonary fibrosis, and asthma. In this review, we summarize how mitochondria morphology, dynamics, redox signaling, mitophagy, and interaction with the endoplasmic reticulum are involved in chronic lung diseases and highlight strategies focused on mitochondrial therapy (mito-therapy) that could be tested as a potential therapeutic target for lung diseases.

## Introduction

Mitochondria are organelles present in all eukaryotic organisms with the classic role of generating most of the cellular energy. Mitochondria are responsible for the synthesis of adenosine triphosphate (ATP) *via* oxidative phosphorylation (OX-PHOS) through the breakdown of sugars and fatty acids in the citric acid cycle (1). In addition to energy production, depending on the cell type, mitochondria can also be involved in other metabolic processes, such as calcium and apoptosis signaling (2–5). Considering their accepted endosymbiotic origin, mitochondria have their own transcriptional machinery, proteome, and DNA (mitochondrial DNA [mtDNA]), which makes them semi-autonomous organelles regulating their homeostasis by autophagy, fusion, and fission (6).

**Figure 1:**
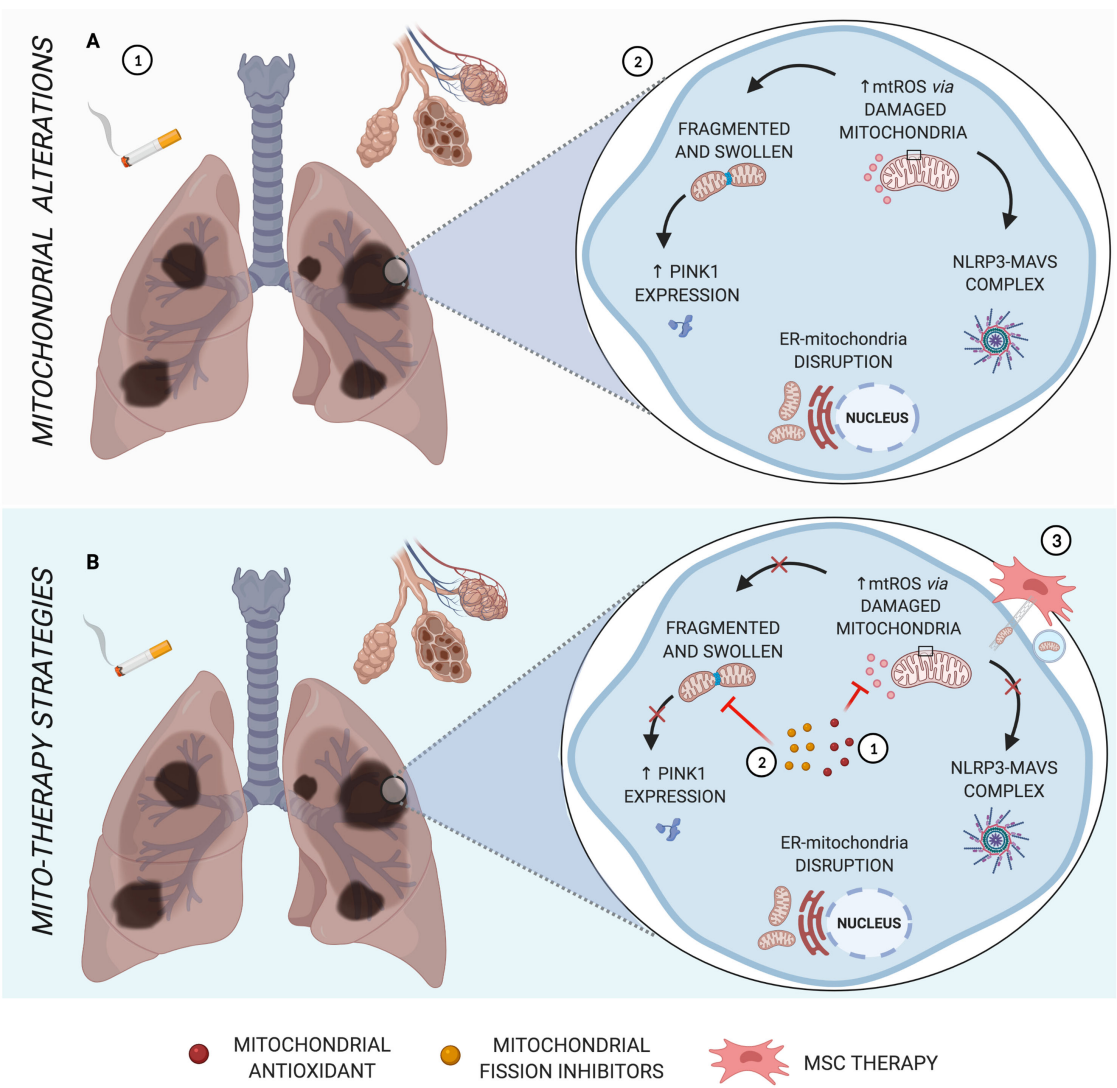
Main mitochondrial alterations in COPD. **(A)** The pathogenesis of COPD, triggered by cigarette smoke, is characterized by alveolar destruction and enlargement, as well as airway inflammation and remodeling (1). As the major source of oxidative stress, mitochondrial dysfunction leads to abnormal morphology, formation of NLRP3-MAVS complex, increased PINK1 mitophagy factor, and disruption of ER-mitochondria crosstalk (2). **(B)** Schematic representation of mito-therapy strategies for COPD. Mitochondrial antioxidants and fission inhibitors have a positive impact on pulmonary cells and murine models of COPD, acting in mtROS (1) and mitochondrial morphology dysfunction (2), respectively, and can act indirectly in NLRP3-MAVS complex formation, innate immune signaling for which mtROS is one activation signal. Otherwise, cell rescue from induced cigarette smoke or oxidative stress occurs *via* iPSC-MSC-mediated mitochondrial transfer in COPD models (3). Created with BioRender.com.

**Figure 2:**
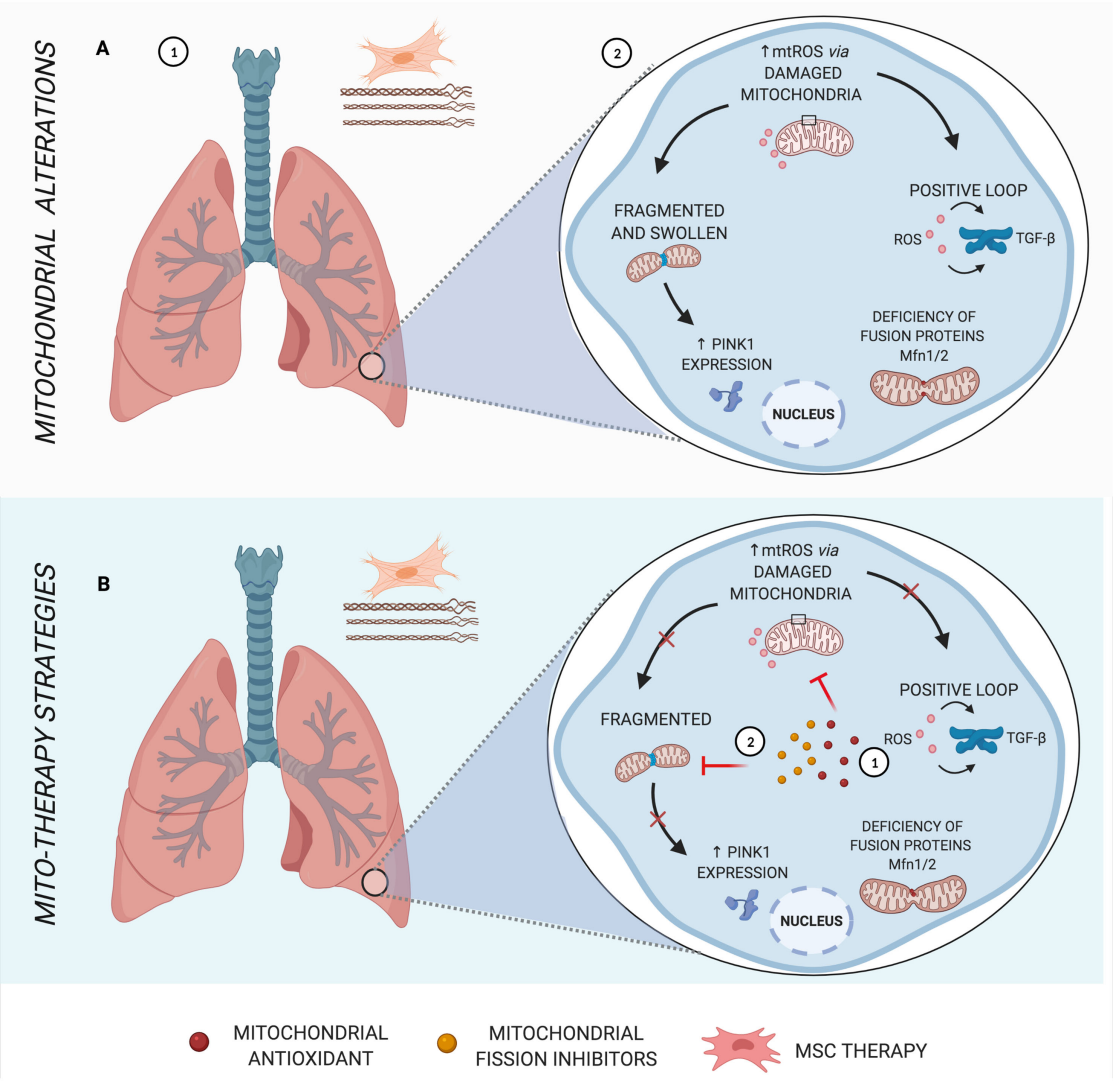
Main mitochondrial alterations in IPF. **(A)** Idiopathic pulmonary fibrosis (IPF), a parenchymal lung disease, is characterized by clusters of fibroblasts/myofibroblasts and excessive deposition of disorganized collagen and extracellular matrix, causing heterogeneous fibrosis (1). Increased mtROS in the pathogenesis of IPF induces transforming growth factor β (TGF-β), stimulating fibrogenesis in a positive loop, in addition to mitochondrial morphology alterations and deficiency in fusion proteins (2). **(B)** Schematic representation of mito-therapy strategies for IPF. Mitochondrial antioxidants lead to control of mitochondrial oxidative stress and, consequently, reduce TGF-β expression and activity (1), whereas mitochondrial fission inhibitors are capable of protecting pulmonary fibrosis models from mitochondrial fragmentation and posterior mitophagy factors (2). Created with BioRender.com.

**Figure 3:**
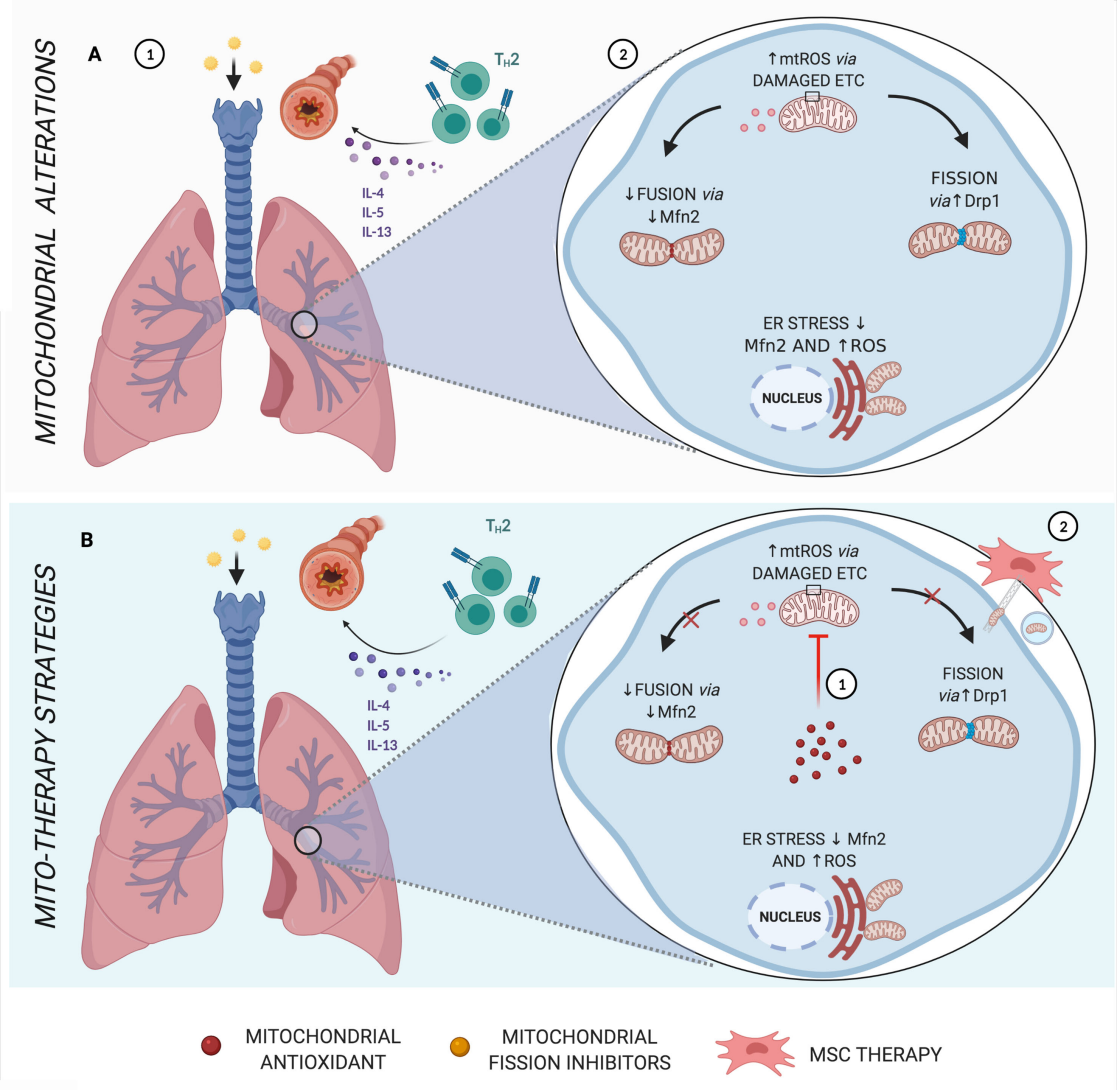
Main mitochondrial alterations in Asthma. **(A)** Asthma, which in most cases is strongly linked to allergen sensitization, is characterized by a Th2 inflammatory response *via* cytokines IL-4, IL-5, and IL-13, leading to bronchial hyperresponsiveness and remodeling (1). Features of asthma have also been associated with increased mtROS, endoplasmic reticulum (ER) stress, reduced fusion proteins, and increased fission dynamics (2). **(B)** Schematic representation of mito-therapy strategies for asthma. Mitochondrial target and localized antioxidants attenuate asthmatic pathophysiologic characteristics, especially controlling mtROS levels (*1*). On the other hand, mesenchymal stromal cells (MSCs) actively transfer mitochondria directly *via* gap junctions or through mechanisms of nanotubes and extracellular vesicles and are associated with beneficial effects in asthma models of airway injury and inflammation (2). Created with BioRender.com.

During homeostasis, mitochondria have important roles in lung function. Mitochondria number and intracellular organization can vary in an energy-dependent form for distinct types of airway epithelial cells (7). Mitochondria can regulate surfactant production, cellular senescence, mucociliary function, and mucus secretion (7). Mitochondria are also crucial in pulmonary immunometabolism and immune cell response, such as in alveolar macrophages (8). Appreciation for mitochondrial noncanonical functions has increased our knowledge of their role in pathophysiologic processes, including chronic lung diseases such as chronic obstructive pulmonary disease (COPD) (**Figure 1**), idiopathic pulmonary fibrosis (IPF) (**Figure 2**), and asthma (**Figure 3**) (9–12).

The centrality of mitochondrial dysfunction in the context of chronic respiratory diseases has been studied extensively in diseases that affect the trachea, bronchi, bronchioles (13, 14), alveoli (15, 16), and interstitium (17, 18). Mitochondrial dysfunction alters cell bioenergetics and hinders lung recovery after an insult (19). Thus, highlighting the importance and challenges of looking at mitochondria as a new target for therapeutic strategies in chronic lung diseases is essential (20–22). This review summarizes some mitochondrial physiologic and pathologic processes, as well as therapeutic strategies for chronic lung diseases considering mitochondria as the central point.

## Mitochondrial Morphology

Structurally, mitochondria differ from other organelles in that their internal compartment (matrix) is kept apart from the cell cytoplasm through inner and outer membranes, separated by an intermembrane space, and all of these components play a fundamental role in their biochemical reactions (23). The conserved inner membrane ultrastructure contains invaginations or cristae where OX-PHOS enzymes, which are fundamental to mitochondrial functionality, are located (1, 24). Moreover, due to their lipid composition, small size, and partial transcriptional independence from the nucleus, mitochondria are extremely dynamic organelles (25). In response to pathophysiologic changes, mitochondria can change structurally and numerically through modifications in the behavior of their protein machinery (26).

In primary type II alveolar epithelial cells (AECII) in patients with COPD, abnormalities in the mitochondrial morphology have been reported, including loss of cristae and swollen and fragmented mitochondria (16, 27, 28). *In vitro* long-term exposure to cigarette smoke extract (CSE) in cultured human airway epithelial cells resulted in similar mitochondrial abnormalities (16, 27). In addition, reduced levels of prohibitin proteins (PHB1 and PHB2), present in the mitochondrial inner membrane, have been observed in lung tissue in patients with COPD and in non-COPD smokers (29). The homologous proteins PHB1 and PHB2 are essential components of fusion machinery and have been found to have a critical role in mitochondrial stability and morphogenesis and more recently in combating oxidative stress (29, 30). Collectively, these data indicate that cigarette smoke (CS) alters mitochondrial structure and functions and downregulates PHB1/PHB2 complexes, leading to increased cellular levels of reactive oxygen species (ROS) and cellular damage (29).

However, mitochondrial changes in COPD are not restricted exclusively to lung parenchyma or airway cells; they also extend to other cell types, such as skeletal muscle cells. Vastus lateralis muscle cells of patients with COPD present reduced mitochondrial fractional area, number, and enzyme activities, resulting in loss of oxidative capacity, which can help to explain the peripheral muscle dysfunction that is a hallmark of COPD (31). In addition, higher rates of apoptosis of T lymphocytes observed among patients with COPD can be partially explained by mitochondrial cytochrome c release, generally associated with abnormalities in mitochondrial morphology (32, 33). Cytochrome c can be released from swollen mitochondria *via* both permeability transition pore-dependent and independent mechanisms, and this liberation is associated with apoptotic cell death (34). T cell apoptosis may be also associated with the high frequency of infections and exacerbations observed among these patients, due to the resulting defective immune response (34).

Mitochondrial structure changes, such as a less dense matrix, loss of cristae, and mitochondrial cavity appearance have also been observed in experimental models of asthma induced by ovalbumin (35, 36). Allergic asthma appears to induce mitochondrial structure changes in an IL-4-dependent form, indicating the potential involvement of inflammatory cells like T lymphocytes, activated mast cells, and basophils in mitochondrial morphology changes in asthma pathogenesis (36). However, the molecular mechanism involved remains undetermined. Intense mitochondrial biogenesis was also demonstrated by a higher expression of activated mitochondria in bronchial smooth muscle (BSM) cells of asthmatic when compared to COPD and control patients (37). This indicates that although both asthma and COPD are characterized by BSM remodeling, a specific mitochondria-dependent pathway is required for BSM proliferation only in asthma (37).

Similarly, experimental models and human samples of pulmonary fibrosis demonstrated an increased number of mitochondria with either a swollen appearance or disorganized cristae in pulmonary epithelial cells (38, 39). Changes in mitochondrial morphology also occur in IPF lung fibroblasts, with disrupted membranes and altered cristae compared with normal subjects (40). Mitochondria modulate cellular senescence, a multifaceted cell phenotype that contributes directly to IPF, partially by increasing mitochondrial biogenesis, many of which become dysfunctional (41). Collectively, these results demonstrated that altered mitochondria morphology is a key pathologic feature of lung fibrosis. Comparatively, less information is currently available regarding modifications in mitochondrial morphology in the physiopathology of asthma and fibrosis compared to COPD.

## Mitochondrial Dynamics

The mechanisms involved in mitochondrial membrane remodeling, termed mito-dynamics, include fusion and fission, which are tightly associated with homeostasis adjustment (42). The fusion of mitochondrial membranes, normally stimulated by energy demand and stress, restores the damaged mitochondria by diffusion and sharing of molecular components between organelles (43). The main GTPases involved in mitochondria fusion are mitofusin 1 (Mfn1) and mitofusin 2 (Mfn2), which are integrated into the outer membrane and have domains exposed to the cell cytoplasm, together with optic atrophy 1 (Opa1), a mitochondrial dynamin associated with the inner membrane (44). On the other hand, fission divides and creates new mitochondria, so it is essential to cell cycle progression through growth and division. The presence of fission machinery is a hallmark and is mandatory for mitosis phases when mitochondria appear to be more fragmented (45, 46). Mitochondrial fission is mediated by a dynamin 1-like (Drp1) and mitochondrial fission 1 (Fis1) protein, but the mechanism has not been fully elucidated yet (47). Alterations in mito-dynamics are observed in many chronic lung diseases and contribute differently to each of them (48).

Mitochondrial dynamics dysfunction has been proposed to participate in the development of COPD (27, 49). CSE directly affects fibroblast, alveolar and small airway epithelial cells, causing substantial mitochondrial morphological defects observed after exposure to non-toxic doses through mechanisms that involve mitochondrial elongation (50, 51). Low doses of CSE also cause increased MFN expression in mouse alveolar epithelial cells (50). In contrast, mitochondrial fragmentation induced *via* Drp-1 recruitment is observed in human bronchial epithelial cells (HBEC) after exposure to more toxic doses of CSE (27, 52). Even more, long-term exposure to CSE causes more complex changes in mitochondrial morphology, reflecting the coexistence of different mitochondrial phenotypes, both elongated and fragmented, to different levels of chronic cigarette smoke in COPD (16, 27).

CS is also known to enhance respiratory disorders such as bronchitis and asthma, characterized by inflammatory changes, hyperresponsiveness, and increased cell proliferation of airway smooth muscle (ASM) (53). ASM cells isolated from moderate asthmatic patients appear to be more sensitive to CSE than non-asthmatic patient samples, with associated decreased expression and function of Mfn2, whereas increased Drp1-mediated mitochondrial fragmentation (49). This mitochondrial fission/fusion imbalance alters ROS dynamics and can lead to a cycle with more fragmented mitochondrial networks, elevated ROS production, and cell proliferation (49). There is still few current information on mitochondrial fission/fusion dynamics in ASM, and its importance in asthma.

Accelerated senescence is observed in lung epithelial cells in IPF, and aging. Alveolar epithelial cells derived from aged mice demonstrated accelerated lung fibrosis with enlarged mitochondria and ​​augmented expression of OPA1 and MFN1/2 (18). This data indicates that mitochondria fusion is predominant in IPF lung epithelial cells (18). In contrast, the absence of mitochondrial fusion proteins Mfn1/2 in murine AECII is strongly associated with less production of surfactant lipids and subsequent spontaneous fibrotic remodeling in the lung, leading to higher morbidity and mortality in these animals (54). Therefore, deficiency in mitochondrial fusion may be linked to disruption in lipid metabolism, AECII injury, and further fibrosis (54). Similarly, the main protein involved in fission, Drp1, has been shown to have critical involvement in the development of pulmonary fibrosis, and when inhibited, it prevents mitochondrial fragmentation and pulmonary fibrosis in a bleomycin-induced model (55, 56). These data suggest that the relationship between mitochondrial dynamics and cell survival/death programming is complicated and may vary between individual cell types and disease conditions (49).

The dynamics of intracellular organization and localization of mitochondria are likely to influence several aspects of cellular physiology. A bidirectional coupling between mitochondrial morphology/dynamics and motility has been proposed as an interconnected signaling pathway involved in cellular function (57). The subcellular distribution of mitochondria can be actively modified in response to energy demand and stress. Organelle dislocation is mediated by cytoskeleton and motor proteins (Miro1/2, actin, microtubules kinesin, and dynein), which can interact with mito-dynamic proteins and can interfere with endoplasmic reticulum (ER) communication (26, 58–61). Mitochondrial intracellular movement is directly linked to calcium signals, which at different concentrations can induce mitochondrial translocation or provide a mechanism to retain mitochondria at Ca^2+^ signaling sites, regulating local power supply (62, 63). This can be particularly important for epithelial cells in chronic lung diseases, such as asthma and COPD, which have a high cell turnover rate and increased energy requirements (20). Other biochemical signals have been involved in the distribution of mitochondria, such as hypoxia, which has been reported to cause mitochondrial translocation to the perinuclear region (64). However, intentional subcellular mitochondrial positioning in chronic lung disease cells and what these mechanisms tell us about mitochondrial function is still a poorly studied topic.

## Mitochondrial Redox Signaling

Together with NADPH oxidases, mitochondria are the major source of ROS, a mitochondrial subproduct generated during the electron transport chain (ETC) flux (65). About 3% of the electrons leak in this process, reacting with oxygen on the mitochondrial matrix to form superoxide (O_2_-), which is converted into hydrogen peroxide (H_2_O_2_) by superoxide dismutase 2 (SOD2) (65, 66). Although ROS are essential for physiologic functions, oxidative/antioxidative imbalance can be detrimental, particularly in organs continuously exposed to oxygen and, consequently, highly susceptible to injury mediated by ROS such as the lungs (67–70).

Mitochondrial ROS (mtROS) can act as a second messenger, promoting physiologic signals of cellular stress and leading to mitochondrial translocation (64, 67). Particularly in the lungs, due to their anatomy and continuous exposure to the environment, mitochondrial respiration is an important endogenous source of oxidative stress (71, 72). When in excess, mtROS leads to uncoupled ETC, calcium imbalance, impaired communication between the ER and mitochondria, and damaged mtDNA, and acts as an inflammatory signal (73).

Mitochondrial damage has an important role in the pathogenesis of COPD, in which ROS levels exceed the antioxidant defenses (74). The abundant ROS production can be explained by CS and CSE lipophilic components were capable of disturbing mitochondrial function and increasing the mtROS in ASM cells from patients with COPD (75). Oxidative stress causes lipid peroxidation, resulting in protein carbonylation, commonly referred to as “carbonyl stress,” that is predominantly associated with chronic diseases (76). In this cycle, carbonyl stress can damage mitochondrial proteins and drive further endogenous production of ROS (69).

Increased mtROS has been demonstrated in a number of fibrotic disorders, including pulmonary fibrosis. Oxidants have a direct impact on the production of the most potent fibrogenic cytokine, transforming growth factor β (TGF-β), inducing its gene expression. The overexpression of this central mediator of fibrogenesis increases the production of mtROS by blocking complex III activity and suppressing the antioxidant system in a reciprocal upregulation (positive loop) (77–79). mtROS also causes oxidation of lipids and proteins identified in bleomycin-induced mouse models of pulmonary fibrosis and in patients with IPF (80, 81). Similarly, exposure to asbestos fibers both *in vitro* and *in vivo* leads to increased mtROS production, which regulates lung epithelial cell apoptosis and fibrosis (82, 83).

Oxidative stress also plays an important role in allergic airway disorders. Airway remodeling and the immune response in asthma pathogenesis have been associated with mitochondrial metabolism, including the redox state (84). The most prominent stimuli of asthma, environmental factors, can lead to damage to specific chain-complex proteins, sustaining ROS generation, and can further lead to airway hyperresponsiveness (AHR) (85, 86). The cellular redox imbalance results in inflammatory infiltration and cell damage and can lead to severe asthma and reduction of the corticosteroid response (87–89). The more severe symptoms in allergic disorders have been associated with mitochondrial defects around complexes I and III, which are responsible for the majority of mtROS production due to electron leakage (85). Several markers of oxidative activity are present in people with asthma. These patients have increased production of ROS by inflammatory cells, such as macrophages, eosinophils, and neutrophils, which lead to an increased concentration of exhaled hydrogen peroxide and secretion of myeloperoxidase and eosinophil peroxidase (87–91).

## Mitophagy

Mitophagy is a selective form of apoptosis for dysfunctional mitochondria, classically through phosphatase and tensin homolog (PTEN)-induced putative kinase 1 (PINK1) degradation (92). Permeabilization of the outer mitochondrial membrane *via* apoptosis regulator Bcl-2 associated X (BAX) and/or Bcl-2 homologous antagonist/killer (BAK), or the opening of the mitochondrial permeability transition pore (mPTP) in the inner mitochondrial membrane leading to the release of intrinsic apoptosis-induced factors, such as cytochrome c, is described to initiate the mitochondrial apoptotic pathway (93, 94). Permeabilization of the outer membrane (MOMP) and activation of fusion and fission mechanisms are necessary to release cytochrome c from cristae junctions (95, 96). Excessive levels of mtROS can induce mitophagy, which in turn removes and recycles toxic or damaged mitochondria, reducing mtROS, to maintain the intercellular balance between oxidants/antioxidants, triggering a negative feedback loop mechanism (97, 98).

Intriguingly, both enhanced and impaired mitophagy have been implicated in the pathogenesis of COPD. *Pink1*-deficient mice showed protection against the main characteristics of COPD, such as airspace enlargement, mucociliary clearance, and mitochondrial dysfunction (99). Accordingly, increased expression of PINK1 in lung epithelial cells of patients with COPD has also been observed, along with increased necroptosis markers, impaired alveolar macrophage autophagy (100), mitochondrial dysfunction, and morphology alteration in skeletal muscle (101). On the other hand, insufficient mitophagy and reduced expression levels of PARK2 (parkin RBR E3 ubiquitin-protein ligase) can accelerate senescence and are part of the pathogenesis of COPD (52). The PINK1-PARK2 pathway has been proposed as a crucial mechanism implicated in mitophagic degradation (102). Mitochondria with depolarized membrane stabilize PINK1, resulting in recruitment of PARK2 to mitochondria, which leads to mitochondrial substrates ubiquitination (102). Concomitant accumulation of ubiquitinated proteins is recognized as at least partly reflecting insufficient mitophagy (103).

PINK1, LC3-I/II, and other mitophagy factors, which are responsible for normalizing mitochondrial morphologic and functional integrity, play a protective role in the pathogenesis of COPD (104). The exposure of pulmonary fibroblasts to CSE led to damaged mitophagy, an increase in cell senescence, mtDNA damage, decreased mitochondrial membrane potential, and ATP levels, later restored by a specific mitochondrial antioxidant (51). These data demonstrate the important role of mitophagy in the pathogenesis of COPD, leading to senescence or programmed cell death depending on the level of damage (52).

In addition, TGF-β can also lead to mitophagy, stabilizing the mitophagy initiating protein PINK1 and inducing mtROS (38). TGF-β is known to stimulate ROS production, and oxidative stress can activate latent TGF-β, setting up a bidirectional signaling and profibrogenic cycle (78, 105). Mechanisms that activate TGF-β-mediated pro-fibrotic events and the PI3K/Akt signaling cascade are important pathways involved in the progression of pulmonary fibrosis (106, 107). In this context, berberine was capable of inhibiting PI3K/Akt/mTOR cascade activation, enhancing autophagy, and mitigating fibrotic markers in a bleomycin-induced rodent model of pulmonary fibrosis (107). PINK1 deficiency was recently correlated with pulmonary fibrosis, and its impaired expression led to an accumulation of damaged mitochondria in lung epithelial cells from patients with IPF (18). *Pink1*-deficient mice are more susceptible to developing pulmonary fibrosis in a bleomycin model, suggesting PINK1 may be necessary to limit fibrogenesis (38). These data together suggest that downregulation of autophagy or mitophagy is deleterious, whereas its upregulation is protective in IPF (108).

Environmental factors and allergens are the main factors involved in the development of allergic airway inflammation and asthma, leading to oxidative stress, mitochondrial dysfunction, and cellular senescence (109–112). Environmental pollutants can induce mitophagy, ROS, and mitochondrial damage, which activate the PINK/Parkin pathway (113, 114). The Ca^2+^/calmodulin-dependent protein kinase II (CaMKII) has been shown to be an important mediator in allergic inflammation, ROS production, and correlated with the severity of asthma (115, 116). Oxidized CaMKII stimulates transcriptional activators of TGF-β and can lead to a profibrotic phenotype, a key factor in the development of asthma airway remodeling (115, 117, 118). Recently, the allergen-ROS-ox-CaMKII-mitophagy axis was demonstrated to play an important role in the development of allergic airway inflammation, indicating that CaMKII may be a therapeutic target for asthma (119). However, studies on mitophagy and asthma are still limited.

## ER Interaction and Calcium Regulation

The ER is responsible for intracellular Ca^2+^ storage; protein synthesis, transport, and folding; lipid and steroid synthesis; and carbohydrate metabolism (120). This organelle interacts with mitochondria through membrane ER contact sites, which involve portions of membrane known as mitochondrial associated membranes (MAMs), which play role in structural and functional linkage for intracellular functions (121, 122). At the MAMs, Ca^2+^ is transferred and can interfere in mitochondrial metabolism, stimulating ETC complexes and regulating ATP production (123).

ER-mitochondria interaction provides a platform for the regulation of mitochondrial dynamics and is related to different pathophysiologic contexts, such as immune response and cell death (124). ER stress, normally caused by unfolded proteins and mitochondrial dysfunction, leads to an increase in ROS production, which, in a vicious cycle, leads to further ER stress (125). However, which mechanism triggers the processes – endoplasmic/sarcoplasmic reticulum stress (ER/SR stress) or mitochondrial dysfunction – is still unclear.

ER-mitochondria crosstalk is disrupted in COPD by stress, such as inhaled tobacco products and pollutants (126). Previous studies have shown increased expression of proteins related to ER stress (chaperones, GRP78, CHOP) in lung cells from mice exposed to CS, bronchoalveolar lavage fluid, and tissue samples from chronic cigarette smokers (127–129). Similarly, AECII injury associated with ER stress markers is a well‐accepted theory in the pathogenesis of IPF (130). Increased mitochondrial content in AECIIs and mitochondrial dysfunction associated with ER stress were found in highly fibrotic areas in IPF lungs (18, 131, 132). Findings in bleomycin-treated mice and AECII of IPF lungs have shown that a disruption in the crosstalk between ER and mitochondria occurs, probably involving mitochondrial homeostasis-control mechanisms, ER stress induced by PINK1, and integrated stress response transcription factors 3 and 4 (ATF3 and ATF4) (130, 133).

ER stress-induced by TNFα and ROS has also been shown to reduce the proteins involved in the connection between ER and mitochondria through MAM, such as Mfn2, in human airway smooth muscle (hASM) cells (134). The exposure of hASM cells to TNFα, a proinflammatory cytokine that mediates the inflammatory response in asthma, led to the activation of ER stress pathways, disrupted mitochondrial proximity to the ER, and decreased Mfn2 protein expression, impairing mitochondrial mobility (134, 135). This creates the possibility of a vicious cycle with reduced Mfn2 expression and altered mitochondrial function (125).

Some aspects involving mitochondrial dynamics and ER interaction *via* MAMs remain enigmatic. However, the mitochondria-ER contact sites role mediating immune responses through facilitation of the NOD-like receptor protein 3 (NLRP3)-inflammasome assembly are well known, including second messenger mechanisms such as mitochondrial ROS (136, 137). These multiprotein complexes are composed of the sensor protein NOD-like receptor family, an adaptor protein with caspase domain (ASC), and a pro-caspase 1 protein. When oligomerized, these complexes sense microbial and damage signals (DAMPs and PAMPs), inducing the active form of IL-1β or IL-18 when activated (138).

Increased mRNA levels of NLRP3 inflammasomes in bronchial tissues and systemically, as well higher levels of IL-18 and IL-1β were found in patients with acute exacerbation of COPD than in smokers (139). Similarly, higher expressions of NLRP3 and IL-1β were found in isolated macrophages and BALF of patients with different phenotypes of asthma, and in animal models of this disease (140–142). Additionally, knockdown Drp1 favors NLRP3 activation in mouse bone marrow-derived macrophages, and Mfn2 protein was required for NLRP3 activation after RNA virus infection to form NLRP3-Mfn2-MAVS complex (143, 144). MAVS (mitochondrial antiviral signaling), also has an important role in the pathophysiology of lung fibrosis in a bleomycin-induced model, through their main expression in pulmonary macrophages, amplifying multiple DAMPs signaling (145). MAVS aggregation is observed in lung tissues from human patients with IPF (146). MAVS is well known to induce antiviral genes, acts as a second adapter to the optimal activity of the NLRP3 inflammasome, contributing directly to IL-1β production without inducing IFN-β expression (146). Considering the close relationship between mitochondria and the ER, and their significant contribution to inflammasome activation and chronic lung diseases, MAMs and NRLP3 may be a potential new therapeutic target.

## Discussion

As highlighted in this review, the contribution of mitochondrial dysfunction in the development of the main chronic lung diseases is unquestionable. All evidence suggests the urgent need and the great potential of therapeutic approaches considering mitochondria as a target.

### Mitochondria as a Target and Localized Antioxidants

Restoration of the cellular antioxidant/oxidant level is a good proposal to protect cells and tissue from oxidative stress-mediated disorders (147, 148). Murine models of ovalbumin (OVA)-induced airway inflammation and hyperresponsiveness have shown attenuated asthmatic lung pathophysiologic characteristics when the redox state was restored using a small-molecular-weight thiol antioxidant compound, N-acetylcysteine amide (AD4) (87, 88). Recently, mitochondrial target antioxidants began to be widely studied as therapeutic approaches to diseases in which oxidative stress appears to be critical (149).

MitoTEMPO was reported as a SOD mimetic antioxidant that inhibits mtROS. It is combined with the lipophilic cation triphenylphosphonium (TPP^+^), a membrane-permeant cation that permits the accumulation of antioxidants inside mitochondria by the membrane potential generated (150). Treatment with mitochondrial-targeted antioxidant MitoTEMPO reduces significant features of asthma in cultured cells and in OVA-challenged mice, suggesting that controlling mtROS levels may reduce TGF-β expression and activity (118). MitoTEMPO also contributes to decelerating fibroblast senescence in patients with IPF (151). However, MitoTEMPO failed to inhibit airway inflammation and bronchial responsiveness in an acute ozone-induced murine model of airway inflammation and bronchial hyperresponsiveness (152). Another mitochondrial-specific antioxidant linked to TPP+, the mitoquinone (MitoQ), is a derivative of coenzyme Q and was capable of reversing mitochondrial dysfunction, inflammation, and AHR after mice exposure to ozone (69, 153). Both MitoQ and Tiron, a mitochondrial localized antioxidant, were effective in inhibiting TGF-β-induced proliferation and CXCL8 release in ASM cells from patients with COPD (69). In addition, SS-31, a mitochondrial-targeted agent, is beneficial in other respiratory conditions such as mechanical ventilation-induced diaphragm weakness and pulmonary arterial hypertension *in vivo* (154, 155). These data reinforce the role of mitochondrial ROS and its potential as a therapeutic approach in lung chronic disease. However, faced with so many types of mitochondrial involvement in pathological processes viewed in this review, the real contribution of mtROS is a question that remains open.

However, antioxidant therapy is still highly questioned for its disappointing results in clinical trials (156). The beneficial effects have been determined only by the pharmacologic properties, ignoring bioavailability and pharmacokinetics, by examining effects in concentrations that are often impossible to achieve *in vivo* (157). Now, antioxidants targeting mitochondria make this therapy more selective and effective, but before it becomes an actual therapy for patients, it is still necessary to carefully establish bioavailability and safety profiles of using more selective agents to achieve clinically relevant effects.

### Mitochondrial Dynamics as a Target

Although mitochondria are very dynamic organelles and changes in fusion and fission are constantly observed in chronic lung diseases, this aspect is often overlooked when considering new therapeutic approaches. Mito-dynamics seems to be an important target because evidence indicates that when disrupted, mitochondrial function is affected negatively (158–160).

Mitochondrial division inhibitor 1 (Mdivi-1) reduces Drp1, Fis1 genes, and consequently excessive mitochondrial fission while enhancing Opa1, Mfn1, Mfn2 genes, and mitochondrial fusion activity (161). Furthermore, Mdivi-1 induced increased levels of complex I, II, and IV enzymatic activities (161). This mitochondrial division/mitophagy inhibitor was capable of reducing CS-induced cell death and mitochondrial dysfunction *in vitro* and protected mice from bleomycin-induced mitochondrial fragmentation and pulmonary fibrosis (56, 99). P110, which is a selective inhibitor of Drp1 enzyme activity and blocks Drp1/Fis1 interaction, was demonstrated to be neuroprotective and improve mitochondrial function and integrity (162). These data suggest that inhibitors of Drp1 might be useful for the treatment of diseases in which excessive mitochondrial fission occurs. Elucidation of mitochondrial dynamics involvement in different cellular processes is promising but still superficial, along with the impact of altered mitochondrial dynamics in chronic lung diseases​​ physiopathology.

### Cell Therapy

Interest in the therapeutic potential of cell therapy in lung biology and diseases has increased (163, 164). This research area is expanding rapidly, and several studies have demonstrated the potential of immunomodulation and regenerative effects of adult mesenchymal stromal (stem) cells (MSCs), in animal models of chronic lung diseases such as asthma, COPD, and fibrotic injuries (165–169). Promising results in animal studies and incipient clinical trials have made MSC therapy further increasingly recognizing the potential contribution of mitochondrial transfer from the MSCs as a potential mechanism of action (170, 171).

Intercellular mitochondrial transfer occurs *via* mechanisms including tunneling nanotube formation between two spatially separated cells, secretion of extracellular vesicles containing mitochondria, gap junctions, and cell fusion where cells will share organelles and cytosolic compounds (172). MSCs can transfer mitochondria to other cells in response to stress signals such as the release of damaged mitochondria, mtDNA, and mitochondrial products along with increased levels of ROS (173). MSC-mediated mitochondrial transfer can have an impact on inflammatory responses and cell viability and is emerging as a therapeutic strategy partially by acting as bioenergetics supplementation (174, 175).

Active mitochondrial transfer from adult stem cells to cells pretreated with ethidium bromide, with defective or deleted mtDNA by mutation, was capable of rescuing aerobic respiration of these nonfunctional mitochondria (175). BMSCs exerted protective effects on the alveolar epithelium, restoring the alveolar metabolism in an acute lung injury (ALI) model. These cells transferred mitochondria to epithelial cells *via* connexin-43 gap junctions, directly or through underlying mechanisms of nanotubes and microvesicles, increasing alveolar ATP production and reducing the hallmarks of ALI induced by lipopolysaccharide (176). Intercellular mitochondrial transport is regulated by Miro1, a calcium-sensitive adaptor protein that helps the mitochondria to move along microtubules inside the cells and when overexpressed, increases their mitochondrial transfer capacity and beneficial effects in asthma models (171). In addition, mitochondrial transfer from human induced pluripotent stem cell (iPSC)-derived MSCs to airway epithelial and ASM cells was also identified, rescuing cells from lung damage induced by CS or oxidative stress (177, 178). iPSC MSCs also attenuate asthma inflammation, protection attributed to mitochondrial transfer *via* connexin 43 (CX43)-mediated tunneling nanotube (TNT) formation (179). Taken together, these data demonstrate that BMSCs can transfer mitochondria and rescue lung damage in different contexts. However, how much of the positive effects of cell therapy in chronic lung diseases are exerted solely by mitochondrial transfer is still unknown.

### Mitochondrial Therapy

Given the observed results with MSC mitochondrial transfer in experimental model systems described above, multiple strategies have been further explored, including local and systemic administration of healthy isolated exogenous mitochondria, also called mitochondrial transplantation or mitoception.

Promising outcomes have been demonstrated in *in vitro* and *in vivo* models. Preclinical studies using New Zealand White rabbits demonstrated cardioprotection in a cardiac ischemia-reperfusion injury after autologous mitochondria transplantation from biopsy samples of the pectoralis major (180). *In situ* mitochondrial injection was capable of enhancing post-infarct cardiac function; mitochondria were internalized by cardiomyocytes 2–8 h after transplantation (180). However, less than 10% of the transplanted mitochondria were integrated into cardiomyocytes (180). Using a similar strategy, systemic intravenously injected mitochondria isolated from cultured human hepatoma cells (HepG2) were used in mice fatty liver models, reducing lipid accumulation and restoring hepatocyte function by less well-known mechanisms (181). Mitochondrial therapy, using isolated mitochondria from C57BL/6J gastrocnemius muscle, has also shown efficacy in a murine model of lung ischemia-reperfusion injury, attenuating lung tissue injury, and mechanical parameters *via* vascular delivery or nebulization (182). More recently, systemic mito-therapy using a mitochondria-rich fraction isolated from BMSCs was capable of decreasing lung, liver, and kidney injury and increased the survival rate in cases of cecal ligation and puncture-induced sepsis (183).

An ongoing trial is testing arterial or tissue injection of autologous mitochondrial transplantation from skeletal muscle of the chest wall into the ischemic myocardium of patients with heart ischemia/reperfusion injury, to decrease morbidity and mortality in patients requiring extracorporeal membrane oxygenation (ECMO) (NCT#02851758). However, it is not yet fully understood if and how mitochondria present in the extracellular space exert effects on cells, and how the internalization of healthy extracellular mitochondria occurs after focal or systemic administration. Remains open in the literature the comparison between the role of MSCs paracrine secretion and mitochondrial transfer.

## Conclusion

Mitochondria-targeted therapy may be a new therapeutic for restoring cellular bioenergetics and function in several airway diseases. Some mechanisms have been acknowledged, demonstrating the complex role of mitochondria in chronic lung diseases. Recent studies have challenged the initial thinking about the central role of mitochondrial oxidative stress, bringing new data about how differently mitochondrial responses can be, acquiring diverse phenotypes in morphology, dynamics, and during mitophagy in distinct diseases. In addition, mitochondria play an essential role in inflammatory signaling, *via* mitochondria-ER communication through MAMs activating NLRP3/MAVS complexes. Therefore, mitochondrial dysfunction was unquestionably a factor in chronic lung disease development and progression. Despite that, innovative and attractive therapy as mitochondrial antioxidants, cell therapy, and mitochondrial transfer remains with important open questions which impact directly their clinical consideration. New insights into these mechanisms may hold the key for mitochondrial target treatment, which has remained elusive.

## Author Contributions

FC, PS, and PR designed this review. All authors contributed equally to literature revision and manuscript writing. All authors contributed to the article and approved the submitted version.

## Funding

Brazilian Council for Scientific and Technological Development (CNPq), Rio de Janeiro State Research Foundation (FAPERJ), Coordination for the Improvement of Higher Education Personnel (CAPES), Department of Science and Technology – Brazilian Ministry of Health (DECIT/MS), and the National Institute of Science and Technology for Regenerative Medicine/CNPq.

## Conflict of Interest

The authors declare that the research was conducted in the absence of any commercial or financial relationships that could be construed as a potential conflict of interest.

## Publisher’s Note

All claims expressed in this article are solely those of the authors and do not necessarily represent those of their affiliated organizations, or those of the publisher, the editors and the reviewers. Any product that may be evaluated in this article, or claim that may be made by its manufacturer, is not guaranteed or endorsed by the publisher.
